# A Personalized Smartphone-Delivered Just-in-time Adaptive Intervention (JitaBug) to Increase Physical Activity in Older Adults: Mixed Methods Feasibility Study

**DOI:** 10.2196/34662

**Published:** 2022-04-07

**Authors:** Jacqueline Louise Mair, Lawrence D Hayes, Amy K Campbell, Duncan S Buchan, Chris Easton, Nicholas Sculthorpe

**Affiliations:** 1 Future Health Technologies Singapore-ETH Centre Singapore Singapore; 2 Saw Swee Hock School of Public Health National University of Singapore Singapore Singapore; 3 Institute of Clinical Exercise and Health Science University of the West of Scotland South Lanarkshire United Kingdom; 4 School of Science, Technology and Health York St John University York United Kingdom

**Keywords:** mobile health, mHealth, sedentary lifestyle, digital health intervention, intervention design, feasibility study, aging, mobile phone

## Abstract

**Background:**

Just-in-time adaptive interventions (JITAIs) provide real time *in-the-moment* behavior change support to people when they need it most. JITAIs could be a viable way to provide personalized physical activity (PA) support to older adults in the community. However, it is unclear how feasible it is to remotely deliver a PA intervention through a smartphone to older adults or how acceptable they would find a JITAI targeting PA in everyday life.

**Objective:**

The aims of this study are to describe the development of *JitaBug*, a personalized smartphone-delivered JITAI designed to support older adults to increase or maintain their PA level, assess the feasibility of conducting an effectiveness trial of the JitaBug intervention, and explore the acceptability of JitaBug among older adults in a free-living setting.

**Methods:**

The intervention was developed using the Behavior Change Wheel and consisted of a wearable activity tracker (Fitbit) and a companion smartphone app (JitaBug) that delivered goal-setting, planning, reminders, and JITAI messages to encourage achievement of personalized PA goals. Message delivery was tailored based on time of day, real time PA tracker data, and weather conditions. We tested the feasibility of remotely delivering the intervention with older adults in a 6-week trial. Data collection involved assessment of PA through accelerometery and activity tracker, self-reported mood and mental well-being through ecological momentary assessment, and contextual information on PA through voice memos. Feasibility outcomes included recruitment capability and adherence to the intervention, intervention delivery *in the wild*, appropriateness of data collection methodology, adverse events, and participant satisfaction.

**Results:**

Of the 46 recruited older adults (aged 56-72 years), 31 (67%) completed the intervention. The intervention was successfully delivered as intended; 87% (27/31) of the participants completed the intervention independently; 94% (2247/2390) of the PA messages were successfully delivered; 99% (2239/2261) of the Fitbit and 100% (2261/2261) of the weather data calls were successful. Valid and usable wrist-worn accelerometer data were obtained from 90% (28/31) of the participants at baseline and follow-up. On average, the participants recorded 50% (7.9/16, SD 7.3) of the voice memos, 38% (3.3/8, SD 4.2) of the mood assessments, and 50% (2.1/4, SD 1.6) of the well-being assessments through the app. Overall acceptability of the intervention was very good (23/30, 77% expressed satisfaction). Participant feedback suggested that more diverse and tailored PA messages, app use reminders, technical refinements, and an improved user interface could improve the intervention and make it more appealing.

**Conclusions:**

This study suggests that a smartphone-delivered JITAI is an acceptable way to support PA in older adults in the community. Overall, the intervention is feasible; however, based on user feedback, the JitaBug app requires further technical refinements that may enhance use, engagement, and user satisfaction before moving to effectiveness trials.

## Introduction

### Background

The importance of physical activity (PA) for healthy aging is well recognized. Alongside a reduced risk of mortality [1], cardiovascular disease [2], and metabolic disease [3], engagement in PA and exercise has been linked with lower levels of depression [4] and elevated quality of life and well-being [5]. Long-term PA has been shown to protect against vascular decline in old age [6], and there is convincing evidence that PA can reduce risk of falls [7] and prevent osteoporosis [8] in older adults. Despite these wide-ranging health benefits, approximately 1 in 4 adults is insufficiently physically active [9]. Given that PA levels tend to decline with age [10], older adults are among the least physically active segments of the population, leaving them at greater risk of chronic conditions and disability.

Interventions designed to promote PA in community-dwelling older adults are effective in increasing PA as well as improving self-efficacy and quality of life [11,12]. Most interventions have incorporated lifestyle counseling and health education elements, typically delivered face-to-face in the home, general practice, or community setting and supported by scheduled remote contact to encourage further involvement in PA [11]. However, such approaches are resource intensive, likely inaccessible to those from remote communities, and cannot fully support the dynamic nature of PA behavior. Furthermore, the recent COVID-19 pandemic has highlighted the need for scalable interventions that do not rely on face-to-face delivery.

Digital health behavior interventions have emerged as a solution to some of these challenges. Early internet-based studies have demonstrated some success in increasing PA and reducing sedentary behavior in both adults [12,13] and older adults [14]. More recently, focus has shifted to mobile health (mHealth) interventions, which offer additional advantages, including on-demand tailored support and the potential for broad reach, scalability, and cost-effectiveness in comparison with face-to-face approaches [15]. Just-in-time adaptive interventions (JITAIs) are a type of mHealth intervention that provide real time *in-the-moment* behavior change support to users when and where it is needed and depending on an individual’s changing needs [16]. The principle is that JITAIs can be more effective than standard mHealth approaches by addressing the dynamic nature of behavior and capitalizing on the changing states of *vulnerability* (need), *opportunity* (namely heightened susceptibility to positive behavior change), and *receptivity* (when someone is able and willing to receive and process just-in-time support) [17]. To do this, JITAIs typically rely on data from the Internet of Things, sensors, connected smartphone apps, or other environmental or contextual inputs.

In the context of PA, JITAIs use activity-tracking data collected through smartphones or wearables to deliver personalized PA prompts at the right time. However, an effective JITAI requires continuous updates from an activity tracker, which represents a significant technical challenge. Currently, only commercially available activity trackers can meet this need. Although activity data can be leveraged from consumer-grade devices such as smartphones, smartwatches, and activity trackers [18], modification of proprietary algorithms is not usually possible. As such, researchers are often forced to develop interventions that match the app’s features, rather than developing an evidence-based intervention and then planning features to align with the intervention [19]. To use data from consumer-grade devices in a theoretically robust mHealth intervention, it is necessary to build a stand-alone but complementary mode of intervention delivery, such as a smartphone app.

Research on JITAIs has, to date, been limited to young or middle-aged populations and community settings such as the workplace, universities, and secondary care [16]; none has targeted older adults in a free-living setting. To address this, as well as the aforementioned issues, we developed a JITAI delivered through a bespoke companion mobile app to help older adults increase or maintain PA levels. This was particularly pertinent because we developed the app in response to the first COVID-19 pandemic–induced lockdown in the United Kingdom, and older adults were identified as being at higher risk from COVID-19 complications than their younger counterparts.

### Objectives

The aims of this paper are three-fold: to (1) describe the development of the *JitaBug* app and intervention, (2) examine the feasibility of conducting a larger definitive trial on the effectiveness of the JitaBug intervention, and (3) explore the acceptability of the intervention among older adults in a free-living setting. We hypothesized that it would be possible to use a commercial app for continuous activity–monitoring purposes, while also being able to deliver our own JITAI, grounded in behavior change theory, with custom messaging, and tailoring variables through our companion app.

## Methods

### Development and Design

The JitaBug intervention was developed during the COVID-19 pandemic in 2020 to maintain or improve PA behaviors in at-risk older populations. The intervention was developed using the Behavior Change Wheel [20], a theory-driven framework based on several models of health behavior that facilitates the systematic development of behavior change interventions. It is underpinned by the *COM-B model*, which is based on 19 existing frameworks of behavior and consists of three necessary conditions for a given behavior to occur: (1) capability, (2) opportunity, and (3) motivation. The development process involved six steps: (1) defining the problem (in this case reduced PA in older adults because of COVID-19–related restrictions), (2) selecting and specifying the target behavior (increasing daily PA levels at home), (3) identifying the COM-B components and psychological determinants to be addressed for behavior change (behavioral diagnosis), (4) using the APEASE (Acceptability, Practicability, Effectiveness, Affordability, Side-effects, and Equity) criteria to identify appropriate intervention functions, (5) selecting appropriate behavior change techniques (BCTs) by using the BCT Taxonomy (version 1) [21] to deliver intervention functions, and (6) identifying the mode of delivery for the intervention. Following these steps, we designed an intervention incorporating a bespoke smartphone app (JitaBug) combined with a wearable activity tracker (Fitbit [Google LLC]) that would allow users to (1) set PA or exercise goals, plan activities, and set reminders; (2) self-monitor PA levels and receive feedback on behavior; and (3) receive just-in-time adaptive prompts (push notifications) with personalized and actionable messages using motivational language to encourage users to meet PA goals. We chose to use a Fitbit device, given Fitbit’s popularity with consumers and therefore the increased potential for future scalability of the intervention and because of the availability of an application programming interface (API), allowing remote data tracking (described in the following sections).

### Intervention Components

Using a combination of self-regulatory BCTs, including goal setting, prompting self-monitoring, providing feedback on performance, and reviewing goals, has been shown to increase the effectiveness of PA behavior change interventions [22].

#### Goal Setting

Goal setting is considered a fundamental component of successful behavior change and is the most frequently used component in health behavior interventions [23]. Evidence from systematic reviews and meta-analyses has shown goal-setting interventions to have small [24] to moderate [25] positive effects on PA. As part of the initial *onboarding* of the JitaBug app, we implemented a goal-setting feature that allowed participants to choose a step count or activity minutes goal depending on their preference. In total, three options were offered—500, 750, or 1000 steps more per day—relative to baseline step count. These targets were informed by evidence that pedometer-based studies typically elicit an increase of 775 steps per day (or effect size of 0.26) in older adults [26]. The activity minutes goal also offered three options—10, 20, or 30 minutes more per day—relative to baseline activity. These goal options were offered to allow participants to gradually increase their PA levels in a realistic and achievable way. Once a goal was selected, participants could rate their level of self-efficacy in achieving their goal by responding to the following question: “On a scale of 1-10, with 1 being not very and 10 being very, how confident are you that you can make some good progress toward your goal?” In addition, participants could review and revise PA goals and reassess their self-efficacy in achieving their goal at any point on the app home screen and were prompted to review their goals every 2 weeks.

#### Planning and Reminders

Action planning, or prompting the user to make specific plans about when and where they will increase their activity, has been suggested as a useful tool to motivate people to change PA behavior [27]. Evidence from systematic reviews has shown action planning to be one of several effective BCTs in increasing both self-efficacy and PA [28,29]. However, experimental evidence concerning the impact of specific BCTs or combinations of BCTs on PA has suggested that action planning only increases PA when combined with coping planning [30]. A planning feature within the JitaBug app allowed participants to plan activities and when and where to perform them. Participants could log activities for a specific date and time, and the app would deliver an activity reminder as a notification. Given that weather is a common barrier to PA [31], a scrollable 7-day weather forecast (presented in 3-hour blocks) was made available on the same planning screen, using the GPS location of the smartphone to assist participants with coping planning; for example, planning indoor or outdoor activities.

#### Self-monitoring and Feedback on Behavior

Self-monitoring is considered an essential technique for PA behavior change and is more effective when combined with one or more other techniques derived from control theory (eg, goal setting, feedback on performance, and reviewing goals) [32]. PA interventions with self-monitoring are more effective than those without and more effective again when combined with action planning and coping planning [30]. We designed the JitaBug app to work alongside a wrist-worn Fitbit activity tracker to allow users to self-monitor PA and progress toward activity goals by viewing summary feedback on the device and on the associated Fitbit app.

We included a *snippets* feature within the app that allowed participants to record voice memos concerning PA behavior using the smartphone microphone. Participants were first asked to indicate (“Yes” or “No”) if they had engaged in PA that day. Depending on the option selected, participants were given guidance on what to record in the snippet, such as the type, duration, and location of PA, as well as reasons for being active or not, feelings about their PA, and any PA barriers experienced.

#### JITAI Messaging

Personalized PA messaging was delivered by the JitaBug app to encourage participants to meet their daily PA goal. Messages were tailored to participants’ context based on (1) real time PA level (from Fitbit data repository), (2) chosen activity goal (step count vs exercise), (3) time of day (anytime, 12:30 PM, 5:30 PM, or 8:30 PM), and (4) good weather versus bad weather from the *OpenWeather* API (good weather defined as <50% chance of rain). A set of 13 decision rules linked to 3 possible intervention options (Table 1) were developed to dictate what message the participant received and when. A total of 136 unique messages were developed (10-14 for each of the 13 decision rules), and depending on whether the participant chose a *step* goal or *activity minutes* goal, they could receive up to 75 unique messages across the intervention period. The time points incorporated into the decision rules were chosen as possibly opportune times to make PA suggestions (ie, to encourage an activity break around mealtimes or to plan an activity for the following day). Once a JITAI message was received by a user’s device, a notification was shown on the lock screen (if locked) or home screen (if unlocked). Clicking on this notification would open the JitaBug app and present the message screen to the user. The message screen displayed the text, any images, and any hyperlinks to resources included in the message. The message screen also included a message-rating question, *Was this useful?,* whereby users could respond by clicking on either a thumbs up or thumbs down image (but not both).

**Table 1:** Just-in-time adaptive intervention option examples.

Scenario		Goal of message	Example		
**Intervention option 1**					
	When a participant meets their daily step or activity minutes goal	Provide positive feedback and encouragement to repeat the behavior	Well done [NAME]! You reached your goal for today! Aim for the same again tomorrow 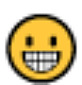 .		
**Intervention option 2**					
	When a participant has not yet reached their daily step goal and the time is between 12:30 PM and 5:30 PM or between 5:30 PM and 8:30 PM and the weather is good	Provide a brief update on goal progress and a message that (1) highlights the benefit of taking an activity break and (2) suggests an activity	You’ve not reached your step goal for today yet, but there’s still time! The weather looks good for this afternoon 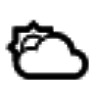 . How about a brisk walk?		
	When a participant has not yet reached their daily activity minutes goal and the time is between 12:30 PM and 5:30 PM or between 5:30 PM and 8:30 PM and the weather is bad	Provide a brief update on goal progress and a message that (1) highlights the benefit of taking an activity break and (2) suggests an activity	Don’t forget that building muscle strength 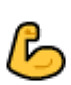 is just as important as aerobic activity; it helps to maintain functional fitness and prevent falls. How many times can you stand up and sit down from a chair during TV ad breaks?		
**Intervention option 3**					
	When a participant did not meet their daily step goal and the time is later than 8:30 PM and the weather is bad	Provide a brief update on goal progress and a prompt that encourages them to plan activity for the following day to meet the goal	The weather is pretty bad tomorrow. But there’s some great online workouts available to try. Does anything take your fancy? [33]		
	When a participant did not meet their daily activity minutes goal and the time is later than 8:30 PM and the weather is good	Provide a brief update on goal progress and a prompt that encourages them to plan activity for the following day to meet the goal	Let’s make sure you reach your step count target tomorrow 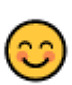 . It’s going to be dry so why not plan something outdoors?		

### Technical Implementation

**Figure 1:**
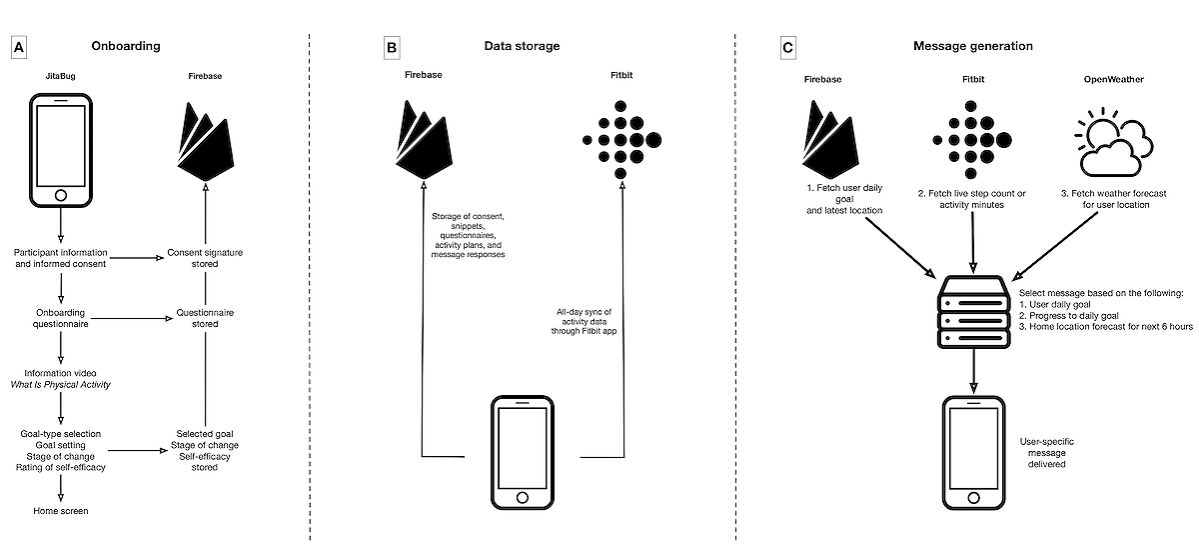
Technical model of the JitaBug intervention, summarizing (A) the onboarding process, (B) how data were stored, and (C) the generation of just-in-time adaptive physical activity messaging.

The technical implementation of the mobile app is depicted in Figure 1. The app was developed using the Dart programming language and the Flutter development framework. This framework has the advantage of enabling apps to be compiled for both Android and iOS platforms with a substantial amount of shared code. Consequently, the final app *JitaBug* was released onto the Apple App Store and Google Play Store shortly before the start of the study intervention (September 2020).

To deliver personalized JITAI messages to each user, we used a separate remote server to automate messaging. This was written using the Python programming language (version 3.7; Python Software Foundation), the Firebase Admin Python package [34], and an open access Fitbit API package [35]. Personalized messages could be compiled by accessing three data sources for each user: (1) a Firebase repository that held data, including the user’s first name, home postal code, chosen activity goals, and mobile phone unique ID; (2) a Fitbit data repository that held the user’s current activity metrics; and (3) the *OpenWeather* API that pulled the weather forecast for the next 4 hours at the user’s home location (based on home postal code). The messaging algorithm ran in 2 parts. Every hour, between 9 AM and 8 PM, the server would check the Fitbit repository to determine whether the user had met their daily activity goal; if yes, it would process and send a motivational *congratulations* message (Intervention option 1; Table 1). In total, three times per day (12:30 PM, 5:30 PM, and 8:30 PM), the server would access the 3 aforementioned data sources and send a personalized message based on the decision rules.

### Feasibility Study Design

We tested the feasibility and acceptability of the JitaBug intervention in a 6-week 1-group pretest-to-posttest trial using a mixed methods approach. The study was conducted entirely remotely across the United Kingdom between September 2020 and November 2020. Participants completed a 7-day monitoring period (baseline), a 4-week intervention (weeks 2-5), and a follow-up 7-day monitoring period. As this was a feasibility study, a sample size calculation was not necessary [36]. Sample size targets were defined by practical and resource considerations, limiting us to recruit 40 older adults.

### Ethics Approval

The study was approved by the Health and Life Sciences Research Ethics Committee of the University of the West of Scotland (13212).

### Participants

Participants were ambulatory, community-dwelling older adults who use a smartphone but not a wearable activity tracker. They were recruited through social media posts from the research teams’ personal accounts (Twitter and Facebook), university newsletter, research recruitment websites [37], community groups (Men’s Shed), and word of mouth. Those interested were emailed a participant information sheet, including contact details for the study team in case they wished to ask questions, and a consent form. After informed consent, participants were asked to provide contact details so that a member of the research team could arrange for the delivery of the study equipment through courier (accelerometer, Fitbit activity tracker, and accessories). In addition, they were sent study enrollment videos and user manuals describing each stage of the study onboarding process.

### Procedure

#### Fitbit Onboarding

Participants were couriered a Fitbit activity tracker (Fitbit Charge 4) for use during the study. The Fitbit trackers were preregistered so that the Fitbit user ID for each device was available to the research team. Notifications were turned off on the Fitbit tracker, the *do not disturb* mode was enabled, and all *exercise* options were removed from the tracker, with the exception of *walk*, before sending to participants to avoid contamination with the JitaBug intervention. Participants were asked to download the Fitbit app and log in using the study username and password provided to them. After logging in, participants were guided to turn off all push notifications and to connect the Fitbit tracker to their smartphone through Bluetooth. They were required to keep Bluetooth turned on for the duration of the study to ensure uninterrupted data upload to the Fitbit data repository (approximately every 15 minutes). This allowed our server to access each participant’s most recent activity data to inform the JITAI decision rules. All participants inputted their date of birth, height (cm), body mass (kg), and sex (male or female) into the *personal* profile section of the Fitbit app.

#### JitaBug Onboarding

**Figure 2:**
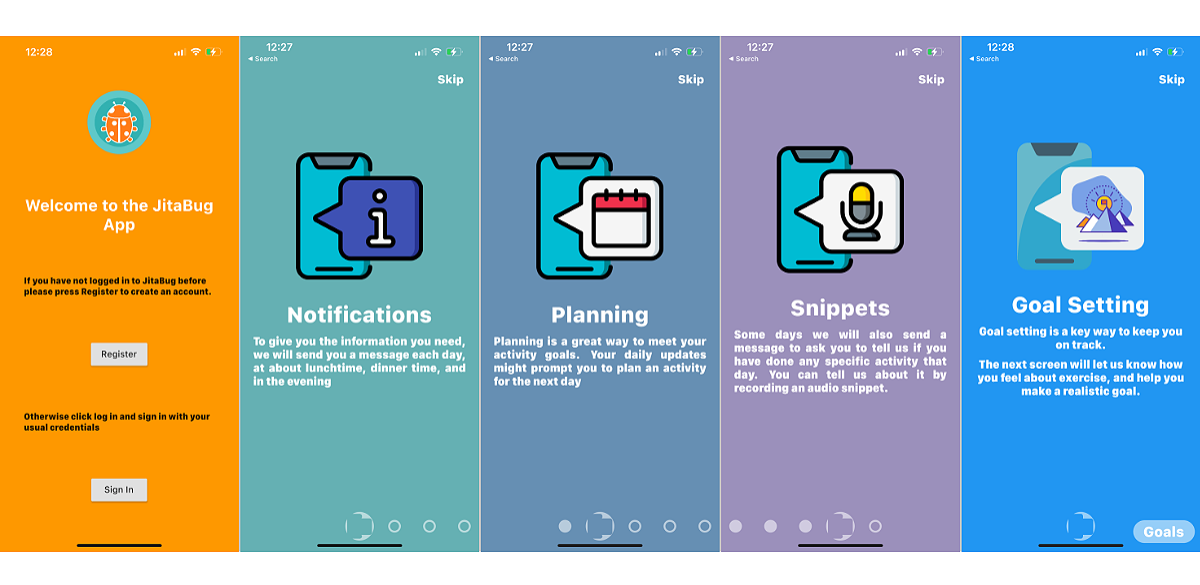
JitaBug onboarding screenshots.

Upon downloading the JitaBug app, participants were asked to reconfirm consent to participate in the research study and log in using the study username and password. Participants then completed a survey to provide personal, anthropometric, demographic, and socioeconomic information. They were then guided through a summary of the app and its key features (Figure 2) including notifications (prompts), planning, the *snippets* feature (described in the following section), and goal setting.

### Main Intervention

After onboarding, participants were directed to the *goal-setting* feature. First, they were shown a short in-app video clip to describe what PA is, what the current PA guidelines are, and how to distinguish between intensity levels. The key message of the video was *any activity is better than none and more is better still* [38]. After they had watched the video, participants were asked to indicate their stage of change [39] with respect to PA from a list of five options:

1. I’ve been physically active for more than 6 months and I am maintaining my activity level (maintenance).
2. I have recently become more active (within the last 6 months; action).
3. I have definite plans to improve my physical activity level in the next month (preparation).
4. I’m seriously intending becoming more physically active in the next 6 months (contemplation).
5. I know I should improve my physical activity level, but I don’t intend to (precontemplation).

Next, participants were shown their average step count and activity minutes from the previous week (baseline). They were then asked to choose either a personal step count goal (500, 750, or 1000 more steps than the average step count during the preintervention period) or an activity minutes goal (10, 20, or 30 more *active minutes* than during the preintervention period) based on their baseline activity level. Finally, self-efficacy in achieving the selected goal was assessed with the following question: “On a scale of 1-10, with 1 being not very and 10 being very, how confident are you that you can make some good progress toward your goal?”

**Figure 3:**
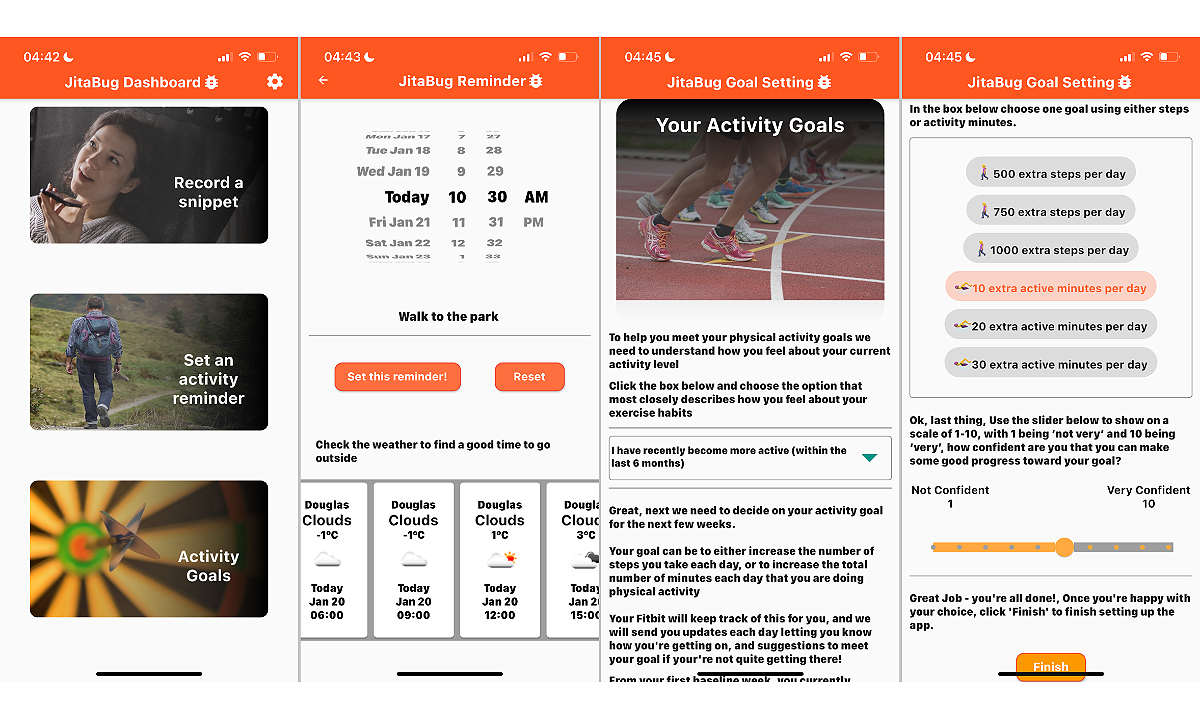
JitaBug app screenshots.

The JitaBug app was designed to mainly run *in the background* with minimal user input. The app automatically delivered PA message prompts (push notifications), in line with the predefined JITAI decision rules, to encourage the participant to meet their goal. Some message prompts encouraged participants to use the activity-planning feature, but they could also access this feature at any point during the intervention. At certain time points throughout the intervention, participants were also prompted to record ecological momentary assessment (EMA) *snippets* to reflect on their progress and describe contextual aspects of their physical activities. After 2 weeks and again after 4 weeks, participants were prompted to review and revise their activity goal or continue with their original goal. Screenshots of the app’s main screens, including the dashboard, planning feature, and goal setting, are shown in Figure 3.

On completion of the study and after safe return of all devices, participants were provided with a £20 (approximately US $26.80) voucher from a reputable retailer as a thank you for their time and effort during the study.

### Outcome Measures

#### Proximal Outcome

The proximal outcome of interest was defined as daily PA goal achievement measured using the Fitbit tracker. The Fitbit tracker was worn throughout the entire study on the nondominant wrist and only removed for charging purposes.

#### Distal Outcome

The main distal outcome of interest was change in time spent in low-, moderate-, and vigorous-intensity PA (minutes per day) and sedentary activities (minutes per day) between baseline and postintervention follow-up. Given the current limitations with wearable activity tracker validity [18] and the need to accurately measure intervention effectiveness in a later definitive trial, we chose to measure PA at baseline and follow-up by research-grade accelerometer. Participants were provided with an ActiGraph wGTX3-BT accelerometer (ActiGraph LLC) and instructed to wear it on their nondominant wrist, distal to the Fitbit tracker when both were worn, for 8 consecutive days, 24 hours per day, during the baseline and follow-up periods, removing it only for bathing or showering. The accelerometers were synchronized with GMT, initialized to capture data at 100 Hz, and programmed to commence data collection at 6 AM on the day after delivery. Participants were instructed to wear the accelerometer on the day it was received to ensure full data capture.

#### Other Outcomes

Sociodemographic and anthropometric characteristics, including gender, age, location within the United Kingdom (postcode), marital status, employment status, key worker status, number of people in the household, household income, education level, number of dependents, dog ownership, bicycle ownership, past activity tracker ownership, and PA preferences, were obtained by means of a survey during the onboarding process to describe the sample recruited.

Self-reported contextual information on PA types, locations, domains, reasons for being active, and barriers experienced was gathered throughout the intervention using the snippets feature that doubled as a voice-based EMA approach. Participants were sent notifications to *record a snippet* at two time points (12:30 PM and 5:30 PM) on two random days of the week (1 weekday and 1 weekend day) each week of the 4-week intervention (16 in total).

We also explored mental well-being and mood responses to the intervention using this approach. On the same day the snippet notifications were delivered, participants were also prompted in the evening (8:30 PM) to complete two questionnaires: the short Warwick-Edinburgh Mental Wellbeing Scale [40] and the short version of the Multidimensional Mood State Questionnaire [41]. The questionnaire screen used a touch interface 6-point (Multidimensional Mood State Questionnaire) or 7-point (Warwick-Edinburgh Mental Wellbeing Scale) Likert scale with a visual indicator of the selected response.

Finally, participants completed a postintervention user experience survey to assess overall experience, app usability and satisfaction with the technology (using relevant questions from the mHealth App Usability Questionnaire [42]), perceived effect on behavior, and views on the intervention as a whole.

### Data Processing

ActiGraph data were downloaded using ActiLife (version 6.14.3; ActiGraph) and saved in raw format as .gt3x files. The files were subsequently converted to time stamp–free .csv files and exported into R (version 3.6.3; The R Foundation for Statistical Computing) for processing using the GGIR package (version 2.1.0) [43]. Briefly, this processing method detected nonwear time as well as abnormally high values and autocalibrated the raw triaxial accelerometer signals using local gravity as a reference [44]. As this was a feasibility study, we were interested in reporting the number of participants who met our proposed wear time inclusion criterion of 4 days, including 1 weekend day, of valid wear (defined as ≥16 hours per day) [45].

### Analysis

The analyses reported here focus on the trial feasibility and acceptability of the intervention. Feasibility outcomes include (1) recruitment and retention within the study; (2) intervention delivery in the wild; (3) completion rates and usable data from the app, the Fitbit wearable activity tracker, and the ActiGraph accelerometer; and (4) adverse events. Results are summarized narratively and descriptively (means, SDs, and proportions) based on data from researcher notes, app analytics, and the user experience survey.

Acceptability outcomes are summarized descriptively based on data from the user experience survey and include three categories: (1) satisfaction with the research overall; (2) satisfaction with the technology (JitaBug app and Fitbit tracker together); and (3) satisfaction with, and usability of, the JitaBug app itself. Likert-scale responses were scored (1=strongly disagree, 2=disagree, 3=neutral, 4=agree, and 5=strongly agree). An overall score for each acceptability category (research overall, technology components, or app components) was calculated by summing the mean score for each question within the respective category. Open-ended responses were coded and categorized into themes. Data are presented as mean (SD), unless otherwise stated.

## Results

### Feasibility

#### Recruitment and Retention

Recruitment advertisements resulted in 75 people contacting the research team about the study. Participants responded through Twitter (2/75, 3%), a research recruitment website [37] (41/75, 55%), existing contact lists (6/75, 8%), or after hearing about the study through word of mouth (26/75, 35%).

After they were provided the study details, 64% (48/75) of the respondents volunteered to participate. Of these 48 volunteers, 46 (96%) met the study inclusion criteria and provided informed consent to participate (exclusion reasons: residing outside the United Kingdom, 1/48, 2%, and current knee injury, 1/48, 2%). Of the 46 participants who consented to participate, 5 (11%) withdrew from the study before receiving the study equipment (Fitbit tracker and accelerometer). Reasons for withdrawal at this point included family emergency (1/46, 2%), other commitments (2/46, 4%), deciding not to take part (1/46, 2%), and unable to download the JitaBug app to an older iPhone (iPhone 5; 2012; 1/46, 2%). Of the remaining 41 participants, 8 (20%) either withdrew or dropped out from the study after having received the equipment. Reasons included no response to study emails/unable to contact the participant (4/41, 10%), issues setting up the JitaBug app (1/41, 2%), did not like the look of the ActiGraph accelerometer (2/41, 5%), and deciding not to take part (1/41, 2%). After completion of the baseline monitoring week, of the 33 remaining participants, 2 (6%) withdrew because of Fitbit syncing issues with their smartphone. Therefore, of the 46 older adults initially recruited, 31 (67%) participants aged 65.5 (SD 5.4) years started and completed the feasibility study. Table 2 summarizes the sociodemographic profile of the final sample.

**Table 2:** Participant characteristics (N=31).

Characteristics						Participants, n (%)
**Sex**						
				Male		14 (45)
				Female		17 (55)
**Location (country in the United Kingdom)**						
			Scotland		20 (65)	
			England		7 (23)	
			Wales		4 (13)	
			Northern Ireland		0 (0)	
**Marital status**						
		Married or cohabiting				21 (68)
		Single				2 (7)
		Widowed				1 (3)
		Divorced				3 (10)
		No response				4 (13)
**Employment status**						
		Employed full time				8 (26)
		Employed part time				1 (3)
		Self-employed				1 (3)
		Retired				16 (52)
		No response				5 (16)
**Education level**						
	Postgraduate					7 (23)
	College graduate or undergraduate					15 (48)
	School					4 (13)
	No response					5 (16)
**Household income,** **£ (US $) per year**						
			<40,000 (<53,651)		2 (7)	
			40,000-59,999 (53,651-80,475)		13 (42)	
			>60,000 (>80,475)		6 (19)	
			No response		10 (32)	
Previous tracker use						4 (13)
Dog ownership						6 (19)
Key worker (performing essential services during the COVID-19 pandemic)						7 (23)
**Stage of change**						
				Precontemplation		1(3)
				Contemplation		0 (0)
				Preparation		8 (26)
				Action		10 (32)
				Maintenance		7 (23)
				No response		5 (16)

#### Intervention Delivery

All participants received the study equipment through courier within 2-5 days of dispatch without issue. All participants were able to download the JitaBug app from the relevant app store (Apple App Store or Google Play Store) independently, but 3% (1/31) of the participants required further assistance from the research team because their device used an operating system that was older than the one the initial design of the app would work with (JitaBug was initially only compatible with Android software development kit version 23 onward). To retain the participant in the study, the research team developed a bespoke version compatible with Android software development kit version 21.

Of the 31 participants, 27 (87%) were successfully onboarded, chose an activity goal, and completed goal self-efficacy at baseline. Goal self-efficacy ratings were generally high (median 8, range 2-10), suggesting that participants were confident in achieving their chosen goal. Of the 31 participants, 14 (45%) reviewed and changed this goal at least once during the intervention period, whereas 4 (13%) required assistance from the research team with onboarding because of lack of mobile data access at the time of onboarding.

Of the 2390 JITAI messages sent throughout the intervention, 2247 (94%) were delivered successfully; of these, 188 (8.37%) were goal-achievement messages from Intervention option 1 (Table 1). To send the right type of message at the right time, the 13 decision rules relied on accurate and up-to-date data from the Fitbit activity tracker and the weather API. Overall, 99% (2239/2261) of the Fitbit, and 100% (2261/2261) of the weather, data calls were successful. Of the 31 participants, 3 (13%) reported that they either “did not receive any notifications” or “received very little information” during the intervention, suggesting that some did not receive this intervention component. These participants were likely those who had difficulty with onboarding because, on investigation, it became apparent that if the process was interrupted or not completed (eg, by closing the app halfway through), then the decision rules would not function because of missing information, namely the activity goal choice. As a result, the participant would not receive any PA messages. The issue was identified and resolved for these participants during the intervention.

#### Data Collection and Missing Data

In terms of accelerometer data, valid and usable data were obtained from 90% (28/31) of the participants at baseline and follow-up. At baseline, of the 31 devices, 30 (97%) were returned for processing, with 29 (97%) files subsequently processed; we were unable to process 1 (3%) device for an unknown technical reason, and 2 (6%) participants failed to meet the minimal wear time criterion. At follow-up, of the 31 devices, 30 (97%) were returned and 30 (100%) files were successfully processed; 3 (10%) files were removed from subsequent analysis for not meeting the minimal wear time criterion. Researcher notes indicated that 6% (2/31) of the participants did not wear the accelerometer while sleeping. The average number of valid days of data was 7.1 (SD 0.5) at baseline and 7.9 (SD 0.7) at follow-up.

Of a possible 496 snippet recordings, 212 (43%) valid recordings were obtained. On average, participants recorded 50% (7.9/16, SD 7.3) of the snippets, 38% (3.3/8, SD 4.2) of the mood assessments, and 50% (2.1/4, SD 1.6) of the well-being assessments through the app. The user experience survey distributed at the end of the study had a 97% (30/31) response rate.

#### Adverse Events

There were no adverse events reported during the study.

### Acceptability

Acceptability data are available in Multimedia Appendix 1.

#### Satisfaction With Research Process

The overall acceptability score for the research process was very good (4.00/5.00, SD 0.73; 80% satisfaction). Most of the participants (27/30, 90%) agreed or strongly agreed that they were satisfied with the research conducted. Most (21/29, 72%) were satisfied with the measurements taken and the amount of data gathered.

#### Satisfaction With Technological Components

The overall acceptability score for the technology components of the intervention was very good (3.86/5.00, SD 0.59; 77% satisfaction). Most of the participants agreed or strongly agreed that they felt comfortable using the technology (21/30, 70%), that it required little effort to use (23/30, 77%), and that it was easy to learn how to use (25/30, 83%). Very few participants agreed that using the technology caused them embarrassment (2/29, 7%). Participants were moderately satisfied with the usefulness of the technology (15/30, 50%) and the accuracy of the data provided by the technology (18/30, 60%).

#### Satisfaction With App Components

The overall acceptability score for the JitaBug app components was good (3.36/5.00, SD 0.72; 66% satisfaction). Most of the participants agreed or strongly agreed that the app was easy to use (22/30, 73%), that the amount of time involved using the app and answering questions within the app was reasonable (18/30, 60%), and that the app’s PA goals were realistic (20/30, 67%). Overall, 59% (17/29) of the participants said that they were satisfied with the app; however, only 43% (13/30) of the participants agreed that they would use the app again. Lower satisfaction (agreement) was also observed with respect to the interface of the app (12/29, 41%), frequency of notifications (16/30, 53%), relevance of notifications (12/30, 40%), usefulness of the information presented within the app (12/30, 40%), and appropriateness of the notification timing (13/30, 43%). Of the 19 questions, 2 (11%) scored high *neutral* responses—satisfaction with the way the app presented feedback and information (17/30, 57%) and satisfaction with the time interval between setting new goals (16/29, 55%)—suggesting that several participants may not have received these aspects of the intervention. Finally, only 33% (10/30) of the participants agreed that the app had all the expected functions and capabilities.

#### Satisfaction With the Intervention

Responses from the open-ended questions of the user experience survey are summarized in Table 3. The feedback identifies potential improvements to the intervention and recommends specific elements that could enhance user engagement in mobile app–based behavior change interventions for older adults going forward. The collective feedback is categorized into themes and links to positive and negative outcomes.

**Table 3:** Summary of qualitative feedback from the user experience survey (N=30).

Theme and positive comments, n (%)			Negative comments, n (%)
**Goals**			
	Motivated to do more, 2 (7)	—^a^	
	Felt good when achieving goals, 1 (3)	—	
**Self-monitoring and feedback**			
	Receiving feedback on behavior, 8 (27)	Fitbit did not track low-intensity activities, 1 (3)	
	Tracking and visualizing behavior patterns, 7 (23)	Unreliable and inaccurate, 1 (3)	
	Comparing with past performance, 4 (13)	Dislike wearing a second watch, 1 (3)	
	Raised awareness, 6 (20)	—	
	Prefer passive data collection, 1 (3)	—	
**JITAI^b^ messages**			
	Took on advice and formed a new habit, 1 (3)	Ignored, 1 (3)	
	—	Annoying, 1 (3)	
	—	Too many, 1 (3)	
	—	Repetitive, 3 (10)	
	—	Irrelevant, 1 (3)	
	—	Too simple or patronizing, 4 (13)	
	—	Inappropriate times, 3 (10)	
**Setting activity reminders**			
	Established a new routine so no longer needed reminders, 1 (3)	Not useful, 3 (10)	
	—	Felt bad when unable to follow through with plans, 3 (10)	
	—	Too time consuming, 1 (3)	
	—	Most difficult part, 1 (3)	

^a^None reported.

^b^JITAI: just-in-time adaptive intervention.

Participants liked using the Fitbit tracker the most (22/30, 73%), followed by receiving feedback on their activity (5/30, 17%). Participants liked using the Fitbit tracker and its associated app to track data on their activity and sleep patterns and visualize their progress over time. This seemed more useful than receiving feedback on behavior through the JITAI messages. Participants felt that the intervention raised their awareness of their PA level and gave them encouragement to be active and meet their goals. There was a suggestion that the passive activity detection offered by the Fitbit tracker was preferred over self-reporting through *snippets*; however, a limitation is that some lower-intensity physical activities are either not detected or do not contribute to steps or activity minutes. Example quotes are as follows:

I think that wearing a tracker automatically makes you more aware of periods of inactivity and progress towards goals, even though it was at times unreliable and didn’t record activities such as tai chi and yoga towards my daily goal.

Participant 2

Fitbit provides immediate feedback on a wide range of health metrics, so enabling more direct tracking of progress, if not against targets, then against historic data.

Participant 4

I was able to see what I was doing and compare days and what my sleep pattern was like. It reported to me on the activity I was doing and my sleep rather than asking me to report what I was doing and then suggest options.

Participant 21

Some participants commented that they found the PA message content to be repetitive and the timing of the messaging was not always appropriate. For example, some reported receiving a message to be active immediately after an activity bout and some mentioned that they were not able to act on the message *in the moment*, which made them feel bad:

They never came in at the right times usually after I had been exercising.

Participant 30

I couldn’t always guarantee to do it and there wasn’t anything I could do about it. So it made me feel bad about myself.

Participant 22

In addition, some mentioned that the language used in the messages was too simplistic and, at times, *patronizing*, and they may be more suited to very inactive people:

...I enjoyed all the information, goals and targets provided with the Fitbit app, so that the JitaBug was really not necessary. One night it did tell me to get out for a walk in the dark, which I did and have continued to do so if I haven’t got my steps in through the day. That was one definite piece of advice from the JitaBug that I responded to. A lot of it was a bit lightweight for me, I feel. I can see that it would be appropriate for some people though (possibly older and less able).

Participant 13

#### Improvement Suggestions From Users

Participants offered several suggestions for improvements to the JitaBug app, predominantly centered around usability. They advised that the PA message notifications should be more obvious (eg, adding a red dot to the app icon on the home screen when a new notification is received) and accessible because messages were not always seen in the notification center. It was suggested that reviewing and setting PA goals should be easier and that there should be more flexibility with the app features (eg, avoid forced completion of EMA questionnaires). Participants suggested that having more content and resources would encourage people to use the app more and that PA messages should be used as an opportunity to provide educational content. Finally, participants suggested that the app should integrate with, and offer more than, existing health apps.

## Discussion

### Principal Findings

This study demonstrates for the first time that a smartphone-delivered JITAI using data from a wearable activity tracker is an acceptable approach to increase PA in older adults in a free-living setting. A main finding is that by using a companion app (and server), it is feasible to leverage the technical benefits of commercial activity trackers and still deliver a theory-led JITAI with bespoke tailoring variables and messaging. Overall, the acceptability of the intervention was very good (23/30, 77% expressed satisfaction). Participants were comfortable with the technology and found the app easy to use. Furthermore, given the intervention completion rate of 67% (31/46), the successful delivery of the intervention *in the wild* as intended, the acceptable levels of accelerometer data collection, and the absence of adverse events, we propose that after some minor usability improvements, the JitaBug intervention is feasible to run in a larger, fully powered trial to ascertain its effectiveness in changing PA behavior and improving health and well-being.

Concerns regarding the acceptability of mobile technology in studies with older adults are largely historical, and our data on the acceptability of the JitaBug intervention confirm what has been reported elsewhere. Hawley-Hague [46] assessed the acceptability of a mobile app to support falls rehabilitation, reporting that older adults had few issues with the technology and were comfortable with using it for exercise advice. Similarly, several studies have reported that older adults find wrist-worn activity trackers acceptable [47,48]. Nevertheless, it is worth noting that the evidence to date is likely subject to sampling bias, given the need for people to own an up-to-date smartphone capable of running new apps and syncing with wearable devices. Indeed, our participants were predominantly well educated, retired, and with reasonable household income. Therefore, more research is needed, across a wider demographic, to understand whether mHealth solutions can truly reach harder-to-reach groups such as those from lower socioeconomic backgrounds and therefore achieve their full potential.

Overall, the JitaBug intervention was well received, the app was easy to use, and participants were comfortable with the technology. However, we noted that participants were less satisfied with the features and content within the JitaBug app and the PA messages in terms of timing, relevance, and repetitiveness. We propose that this was partly due to disparity between the study aims and participants’ expectations of the app. The JitaBug app was designed to run in the background with minimal user input to reduce user burden and limit intervention fatigue [17]. However, participants reported that they wanted more content and interaction and felt that they often had little reason to open the JitaBug app. We intended to avoid recreating the Fitbit app; therefore, we did not include updates on activity minutes or step count within JitaBug. However, to ensure fidelity to the JITAI messaging and BCT approaches, we also requested participants turn off notifications from the Fitbit app. Consequently, participants felt that the JitaBug app offered insufficient information regarding progress toward their goals each day. Future studies should address this and include some feedback on activity data within the companion app if the Fitbit (or another proprietary) app might compromise study fidelity.

Regarding timing, relevance, and repetitiveness of PA messages, specific tailoring variables within decision rules may have influenced delivery of appropriate messages. For example, time lags between the Fitbit wearable activity tracker syncing with the JitaBug servers may have resulted in incongruence between real time PA and the message received concerning current PA. The JitaBug app called the Fitbit server every hour, but this was dependent upon an internet connection and the frequency with which the phone synced data with the Fitbit server. Another variable that could influence message content was weather conditions. Given that the study was delivered in the autumn in the United Kingdom when average rainfall is at its highest and the decision rules determined good versus bad weather depending on rainfall, it is likely that far fewer message options were delivered than intended. Thus, the geographical location and season in which an intervention is delivered may have profound implications for intervention delivery and user acceptability. Finally, we used predefined windows of time, chosen as possible opportune moments to intervene on PA behavior, to send JitaBug notifications. However, event-based timing, for example, when someone has been sedentary for a prolonged period of time, might be a more effective approach [17]. Furthermore, the timing of JITAI messages may be enhanced by detecting a user’s state of receptivity [49] with additional data inputs such as location, device interaction, and battery level [50]. Future studies should expand on the number and type of tailoring variables relevant to PA, which in turn will facilitate the development of more intuitive and nuanced messaging rules.

We anticipated at the outset that creating PA messages that are equally meaningful to participants with different backgrounds, reading abilities, and expectations was likely to be one of the most challenging parts of the study. The language used in our messaging was aimed at a reading level equivalent to 6th grade (12 years of age) in line with guidance on the reading level of patient education and health literacy materials [51]. Nevertheless, participants commonly described the messages as overly simplistic and occasionally patronizing. The same feedback has been reported in another JITAI study targeting substance abuse in adolescents and young adults [52]. Developing meaningful messages is complex, multidimensional, and requires the incorporation of a variety of concepts and theories [53]. It is possible that co-designing messages with intended users might increase satisfaction with this aspect. Moreover, it may be possible to target messages to specific user groups, for example, based on demographics or further tailor messages for individual users based on preferences, baseline activity levels, and even personality traits [54]; however, this would inevitably require significantly greater resource commitment.

In terms of feasibility, web-based, email, and referral recruitment strategies were successful in reaching 75 people over a 9-week period, with 48 (64%) of them then volunteering to participate. Of the 46 volunteers who were eligible, 31 (68%) enrolled onto the study, suggesting a good level of interest in the intervention. These recruitment and retention findings are similar to other PA-focused mHealth studies using apps and commercially available activity trackers in adults [55,56]. Given that the apps used in these previous studies have undergone extensive testing and development over several years to maximize engagement, finding comparable recruitment and retention rates with the JitaBug intervention is promising and further supports the feasibility of the intervention. Furthermore, this study was conducted in the context of COVID-19 restrictions, which required us to develop an intervention that could be delivered wholly remotely. Thus, unlike earlier work, our retention data did not benefit from face-to-face induction of participants onto the study. Indeed, critical aspects of the intervention, including the aims and functionality, which might usually be undertaken by the research team, had to be incorporated into the app *onboarding* process. Nevertheless, retention was relatively unaffected; therefore, the encouraging recruitment and retention data suggest that a high proportion of older adults can feasibly use the JitaBug app remotely.

The feasibility of PA data collection by accelerometer and wearable activity tracker was excellent. We also assessed the feasibility of collecting longitudinal changes in participants’ mood and well-being using an EMA-based questionnaire and contextual information about PA behavior using a voice-based EMA approach, both integrated within the JitaBug app. According to a recent systematic review of smartphone-based EMAs [57], this is the first study to combine qualitative data collection with an EMA approach and only the second to evaluate the feasibility of EMA in older adults. de Vries et al [57] report that although compliance in EMA studies is infrequently reported, it ranges between 43% and 91%, with longer studies trending toward lower compliance. Our compliance of between 38% and 50% is relatively low and is perhaps not surprising, given the length of EMA collection. Nevertheless, our qualitative EMA approach through audio snippets provides a novel and feasible way to gather contextual information about PA behavior, which is currently lacking in device-based PA measurement studies [18]. It is also worth noting that additional reminders when participants miss EMA recordings have not previously been feasible because researchers had to wait until the end of the study to retrieve the EMA data. However, we have demonstrated herein that it is feasible to remotely collect and store snippets, making them immediately accessible by researchers; therefore, EMA reminder notifications are possible and could further increase the feasibility of this data collection method.

### Limitations

There are several limitations of this study that should be noted. Our proximal outcome was achievement of daily PA determined by the Fitbit tracker. This was because the JITAI component of this study was dependent on continuous activity–updating of Fitbit servers to enable remote tracking, a feature not available on research-grade accelerometers. Although validity and reliability of commercial activity trackers for step counting in older adults have been reported as good to excellent [58,59], they are still considered less valid than research-grade accelerometers in measuring PA of different intensities [18,60]. We sought to overcome the limitations with the measurement device by simultaneously determining the feasibility of remote deployment and recovery of research-grade accelerometers in the week before and the week after the JITAI. The accelerometer data loss of 10% (3/31) is encouraging, suggesting that it is feasible to measure PA using ActiGraph in community-dwelling older adults; therefore, this approach can be used to strengthen the validity of efficacy data. A second limitation is that muscle-strengthening activities are not accurately captured through device-based measurement, particularly when step counting is used over heart rate–based activity goals [61]. The importance of muscle-strengthening activities in addition to aerobic PA has been well established, but fewer older adults meet the daily recommendations [62]. Thus, including a means for recording muscle-strengthening activities in the JitaBug app is an area for consideration. Third, we did not collect location data or use other contextual data (such as calendar appointments, device use, and battery status) within our decision rules. These data may improve the timing of, and receptivity to, JITAI messages [50] and should be considered in future versions of the intervention. Finally, the participants recruited were predominantly well educated, retired, and with reasonable household income; therefore, the feasibility of the intervention in other population groups, including those from lower socioeconomic backgrounds, may be required before implementation.

### Conclusions

This study demonstrated that a smartphone-delivered JITAI using a wearable activity tracker (JitaBug) is an acceptable way to support PA in older adults in a free-living setting. Moreover, the intervention was feasible, although the app will undergo further technical refinements that may enhance use, engagement, and user satisfaction before effectiveness trials. Finally, we present a novel and feasible approach to capture qualitative insights into PA behavior alongside quantitative measurement, which may advance the PA measurement capabilities of future smartphone-delivered mHealth approaches.

## Appendix

Multimedia Appendix 1 Acceptability data.

## Abbreviations

APEASE: Acceptability, Practicability, Effectiveness, Affordability, Side-effects, and Equity
API: application programming interface
BCT: behavior change technique
COM-B: capability, opportunity, motivation–behavior
EMA: ecological momentary assessment
JITAI: just-in-time adaptive intervention
mHealth: mobile health
PA: physical activity
