# Supplementary material for: A Personalized Smartphone-Delivered Just-in-time Adaptive Intervention (JitaBug) to Increase Physical Activity in Older Adults: Mixed Methods Feasibility Study
Source: JMIR Form Res. 2022 Apr 7;6(4):e34662. doi: 10.2196/34662 (PMC9030994; doi:10.2196/34662)
Supplement: Multimedia Appendix 1 [file formative_v6i4e34662_app1.docx]

## Multimedia Appendix 1. Acceptability data

Figure 1. Responses to survey questions on technological components of the intervention. *reverse scoring used.

Figure 2. Responses to survey questions on app components of the intervention.
